# A Comparative Study of Dynamic Hip Screw Versus Multiple Cannulated Compression Screw Fixation in Undisplaced Intracapsular Neck of Femur Fractures

**DOI:** 10.7759/cureus.31619

**Published:** 2022-11-17

**Authors:** Samuel E Cullen, Benjamin Sephton, Isfand Malik, Ameer Aldarragi, Martin Crossdale, Michael O’Connor

**Affiliations:** 1 Trauma and Orthopaedics, Stepping Hill Hospital, Stockport, GBR

**Keywords:** cannulated cancellous screw, dynamic hip screw fixation, neck of femur fracture, intracapsular fracture, fracture around hip, hips

## Abstract

Background

Femoral neck fractures are common injuries. Although many studies have compared two-hole dynamic hip screw (DHS) versus multiple cannulated cancellous screw (CCS) fixation for undisplaced intracapsular fractured neck of femurs (NOF), there is no consensus on which surgical technique results in better outcomes. The aim of our study was to compare DHS and CCS for undisplaced NOFs. The primary outcomes were reoperation and mortality rates at one year postoperatively.

Methodology

A retrospective analysis was performed involving all patients who underwent fixation with DHS/CCS for an undisplaced intracapsular NOF at our hospital between January 2016 and December 2020. All patients had a minimum follow-up time greater than one year. All patients underwent a standardised NOF protocol. Patients either underwent DHS or CCS fixation according to surgeon preference, and there was no randomisation to either group.

Results

A total of 41 patients underwent fixation with DHS compared to 32 who underwent CCS. The reoperation rate at one year was 9.8% in the DHS group compared to 6.3% in the CCS group. This difference was not statistically significant (p = 0.588). The one-year mortality rate was similar between groups at 22.0% and 21.9% in the DHS group and the CCS group, respectively (p = 0.994). Registrar-level surgeons performed 80.5% of DHS compared to 59.4% of CCS, and consultant surgeons performed 4.9% of DHS compared to 25% of CCS.

Conclusions

There was no significant difference in revision rates or complications seen between CCS and DHS. A significantly higher percentage of CCS fixations were performed by consultant-grade surgeons at our hospital compared to DHS. This study provides further data on the choice of fixation method for intracapsular fractures. It also reports on the grade of the operating surgeon at our unit, which may be a factor in the quality of fixation and ultimately reoperation rates.

## Introduction

Femoral neck fractures are common injuries presenting to orthopaedic departments worldwide. In the United Kingdom (UK) alone, over 300,000 patients suffer a hip fracture annually. The cost associated with the treatment and aftercare of these injuries is around £2 billion [1]. Globally, populations are ageing, resulting in an increased incidence of hip fractures. Hip fractures are associated with many complications, including non-union, avascular necrosis (AVN), hospital-acquired infections, metalwork failure, and death [2]. With an increasing prevalence of femoral neck fractures, the importance of effective surgical management to minimise associated complications is paramount.

The Garden Classification divides intracapsular hip fractures into four types based on the degree of fracture fragment displacement [3]. Type I and II fractures are non-displaced, while types III and IV are displaced fractures. In the UK, internal fixation is a common treatment modality for non-displaced intracapsular neck of femur (NOF) fractures. The dynamic hip screw (DHS) and percutaneous insertion of three cannulated cancellous screws (CCS) are the most common methods for the fixation of these fractures. Although several studies have compared DHS versus multiple CCS fixation [1,2,4-8], there is no consensus on which surgical technique results in better outcomes.

CCS fixation has the advantage of being able to provide antirotation and antistress stability [4]. CCS is also less invasive, with less soft-tissue stripping than DHS [2,9]. Another recent study showed that when compared with DHS, CCS resulted in shorter inpatient stays and lower rates of 90-day mortality [1]. The problems CCS present, however, are weak anchorage, especially in osteoporotic bone, which can present with early implant loosening [9,10]. DHS fixation can provide more stable fixation in osteoporotic bone and maintain both a normal neck-shaft angle and anatomical fracture reduction [2,4,9]. DHS fixation has been shown to have improved biomechanical stability with respect to CCS fixation, especially with vertical shear-type fracture patterns of the femoral neck [11]. Complications of both DHS and CCS are non-union, infection, AVN, and implant failure with a subsequent need for revision surgery [4,8]. A large-scale randomised controlled trial in 2017 found no significant difference in reoperation rates between DHS and CCS in general; however, it showed that those with displaced or basicervical fractures, or those who smoke, had a significantly lower reoperation rate with DHS fixation [12].

The aim of this local retrospective study was to compare DHS to CCS for fixation of non-displaced (Garden I, II) intracapsular NOF fractures. The primary outcomes were reoperation and mortality rates at one year post-operatively.

## Materials and methods

A retrospective analysis was performed among all patients sustaining an undisplaced NOF fracture (Garden’s type I/II) during the study period from January 2016 to December 2020. This was a retrospective study looking at patient outcomes from standard treatment only. There was no randomisation or change to clinical care due to the study and so ethics committee approval was not required.

The inclusion criteria were as follows: (1) Garden type I and II NOF fracture; (2) patients undergoing operative intervention with two-hole DHS or cannulated compression screws; and (3) follow-up time greater than one year. Exclusion criteria were as follows: (1) age less than 50 years; (2) pathological fracture of the femoral neck other than osteoporosis; (3) previous NOF fracture; and (4) open reduction performed intraoperatively. Identification of suitable patients was performed using Bluespier Trauma Software (Bluespier, UK).

Patients who satisfied inclusion and exclusion criteria had their notes reviewed to determine the following information: patient age, gender, American Society of Anesthesiologists (ASA) grade, comorbidities, and laterality of procedure. To further assess fitness for anaesthesia the Charlson Co-morbidity Index was calculated for each patient [13]. Time to surgery from injury and delay greater than 36 hours were documented. Operation notes were reviewed to determine operative technique, tranexamic acid (TXA) usage, intravenous (IV) antibiotic usage, and the grade of the operating surgeon.

All patients underwent a standardised NOF surgical protocol. Patients were placed in the supine position on a traction table following the administration of anaesthesia. Reduction and position were confirmed using a traction table under image intensification. Two doses of IV antibiotics (flucloxacillin/teicoplanin + gentamicin) and 1 g IV TXA were given at induction with a further 1 g IV TXA at six hours postoperatively. Patients then underwent fixation with a 135-degree two-hole DHS or three 6.5 mm partially threaded CCS depending on the surgeon’s preference. Postoperatively, patients underwent routine physiotherapy and rehabilitation at our institution with attempted mobilisation on postoperative day zero or one. Low-molecular-weight heparin (enoxaparin) was provided for 28 days postoperatively unless established on long-term anti-coagulation which was restarted as per hospital protocol.

Primary outcome measures of reoperation rate and mortality within one year were recorded. Radiographic imaging was cross-referenced across multiple sites in the Northwest Deanery via the SECTRA imaging platform (SECTRA Medical, UK). Only reoperations due to technical failures were included: fracture displacement, non-union, AVN, periprosthetic fracture, and cut-out of implants.

Scale variables with normal distribution are presented as mean (standard deviation, SD). Non-parametric variables and scale variables without normal distribution are presented as median (interquartile range, IQR). The normality of distribution was tested using the Shapiro-Wilk test. Proportions are presented as a number (%). Univariate comparative analysis of scale variables with normal distribution was performed using an unpaired, two-tailed t-test. Analysis of non-parametric variables or non-normally distributed scale variables was conducted using the Mann-Whitney U test. Categorical data were analysed using the chi-square test. All statistics were calculated using XL Stat (Addinsoft, New York, USA). A p-value of <0.05 was considered statistically significant.

## Results

**Figure 1:**
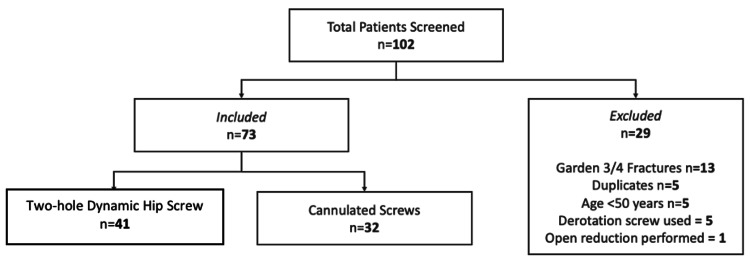
Flowchart showing the breakdown of the patients included in the study according to the inclusion and exclusion criteria.

A total of 102 patients were identified for potential inclusion in the study. Following the application of inclusion and exclusion criteria, a total of 73 patients were included for further analysis (Figure 1). In total, 41 patients underwent fixation with a two-hole DHS and 32 underwent fixation using three CCS. Both study populations were well-matched in terms of patient demographics (Table 1). The male:female ratio in the DHS study group was 1:3.6 versus 1:1.7 in the CCS study group; however, this was not statistically significant (p = 0.145). The average age was 79.1 years (SD = 10.4) and 74.7 years (SD = 12.0) in the DHS and CCS groups, respectively (p = 0.104). No significant differences were found between the ASA scores or the Charlson Co-morbidity Index in both study groups.

**Table 1:** Patient demographics. *: chi-square test; ^±^: unpaired, two-tailed t-test; ^¥^: Mann-Whitney U-test

		Two-hole dynamic hip screw, n (%)	Cannulated compression screws, n (%)	P-value
Gender	Male	9 (22.0)	12 (37.5)	0.145^*^
	Female	32 (78.0)	20 (62.5)	
Mean age (standard deviation)		79.1 (10.4)	74.7 (12.0)	0.104^±^
American Society of Anesthesiologists Score	1	4 (9.8)	1 (3.1)	0.266^*^
	2	7 (17.1)	9 (28.1)	0.257^*^
	3	19 (46.3)	14 (43.8)	0.825^*^
	4	11 (26.8)	8 (25.0)	0.860^*^
Charlson Co-morbidity Index, median (interquartile range)		5 (2)	4 (4)	0.238^¥^

No significant difference was found in terms of the laterality of the procedure (p = 0.779) or type of fractures according to Garden’s classification (p = 0.725) (Table 2). The time to surgery was 40.1 hours (SD = 32.2) in the DHS study group versus 44.4 hours (SD = 53.6) in the CCS group (p = 0.672). Overall, 31.7% of the patients (n = 13) had a delay to surgery of >36 hours in the DHS group compared to 34.3% of patients (n = 11) in the CCS group, which was not statistically significant (p = 0.810). Table 2 also demonstrates that significantly more operations were performed by registrar-grade clinicians for DHS procedures than CCS at 80.5% compared to 59.4% of operations, respectively (p = 0.048). Conversely, a significantly higher proportion of operations were performed by consultant-grade clinicians in the CCS group (25%) compared to the DHS group (4.9%) (p = 0.013).

**Table 2:** Injury and operative details. *: chi-square test; ^±^: unpaired, two-tailed t-test

		Two-hole dynamic hip screw, n (%)	Cannulated compression screws, n (%)	P-value
Laterality	Right	23 (56.1)	19 (59.4)	0.779^*^
	Left	18 (43.9)	13 (40.6)	
Garden’s classification	Type 1	15 (36.6)	13 (40.6)	0.725^*^
	Type 2	26 (63.4)	19 (59.4)	
Mean time to surgery (standard deviation)		40.1 (32.2)	44.4 (53.6)	0.672^±^
Delay to surgery (>36 hours)		13 (31.7)	11 (34.3)	0.810^*^
Grade of operating surgeon	Senior House Officer	6 (14.6)	5 (15.6)	0.907^*^
	Registrar	33 (80.5)	19 (59.4)	0.048^*^
	Consultant	2 (4.9)	8 (25.0)	0.013^*^

In terms of our outcome measures, the reoperation rate at one year was 9.8% (n = 4) in the DHS study group. Reasons for reoperation included one patient with AVN, one patient with non-union, and two patients with the cut-out of the prosthesis (Table 3). The reoperation rate at one year was lower in the CCS group at 6.3% (n = 2); however, this was not significantly significant (p = 0.588). Both patients in this group required reoperation due to the cut-out of the prosthesis. The one-year mortality rate was similar between both groups at 22.0% versus 21.9% in the DHS group and CCS group, respectively (p = 0.994).

**Table 3:** Primary outcome measures following each method of fixation. ^±^: unpaired, two-tailed t-test

	Two-hole dynamic hip screw, n (%)	Cannulated compression screws, n (%)	P-value
Reoperation rate	4 (9.8%)	2 (6.3%)	0.588^±^
Mortality rate	9 (22%)	7 (21.9%)	0.994^±^

## Discussion

This study found a trend towards lower revision rates for CCS fixation at 6.3% compared with 9.8% for DHS; however, this was not statistically significant. This is in line with several recent studies and meta-analyses which have shown no significant difference between the two methods in terms of reoperation rate or mortality [2,7,8,12]. The revision rates at 24 months in a large-scale randomised controlled trial were 22% and 20% for CCS and DHS, respectively. Our revision rates are lower than this, but the follow-up period was also 12 months shorter [12]. The one-year revision rate in our study for both CCS and DHS is higher than seen in a study by Jetoo and James examining over 50,000 patients, for which the revision rate was 3.1% for CCS and 2.0% for DHS [1]. The Jetoo and James study also found the revision rate in CCS to be significantly higher at both one and four years compared to DHS [1].

The most common complication requiring revision was implant cut-out, with a non-significant higher cut-out rate at one year in CCS of 6.3% compared to 4.9% for DHS. The next most common complications were AVN and non-union, which were only seen in the DHS group, both at a rate of 2.4% at one year. The rates of complications requiring revision were again comparable to most recent studies, which also showed no significant difference in complication rates between groups [7,8].

The mortality rate of patients within both groups was very similar at one year, with a mortality rate of 21.9% in the CCS group and 22% in the DHS group (p = 0.994, χ^2^ test). Recent literature appears to show no difference in mortality between CCS and DHS groups [6,7], including the findings of two large meta-analyses [2,8], with the exception of the Jetoo and James study which showed a higher 90-day mortality in patients treated with DHS [1].

A significantly higher percentage of DHS fixations were performed by registrar-grade clinicians (80.5%) compared to CCS (59.4%), corresponding with a significantly lower percentage of DHS being done by consultants compared to CCS (4.9% vs. 25.0%, respectively). This may demonstrate that registrars are more familiar with the technique of DHS fixation, with them being used for extracapsular fractures as well, while CCS is seldom performed during the early years of training. The revision rates at one year were not adjusted for this discrepancy in this study, and this is one theory for our higher revision rate for DHS than CCS, which is contrary to the findings of lower DHS revision rates in the Jetoo and James study [1], There is, however, no data on the grade of the operating surgeon in the Jetoo and James study to compare.

Our study did not have any randomisation to either DHS or CCS groups, meaning that there may be a possible inherent bias in the selection of the surgical technique dependent on fracture pattern, age, comorbidities, functional status, or other variables. This study is also a retrospective analysis which is a limitation.

## Conclusions

This retrospective study examined the possible differences in reoperation rates and mortality rates between patients managed with DHS or CCS for non-displaced intracapsular NOF fractures. We found no significant difference in reoperation rates and mortality rates between the two groups. This study’s findings support previous literature that there is no difference in reoperation rates and mortality rates at one year following either DHS or CCS fixation. The rates of reoperation and mortality at our hospital are comparable with previous studies. A significantly higher percentage of CCS fixations were performed by consultant-grade surgeons at our hospital compared to DHS, which may be a factor in the quality of fixation and ultimately reoperation rates.
